# The Potential of Cannabidiol for Treating Canine Atopic Dermatitis

**DOI:** 10.3390/vetsci12020159

**Published:** 2025-02-12

**Authors:** Ana F. Bizarro, Vanessa M. Schmidt, Beatriz Fernandes, Marta Pinto, Hugo Pereira, Joana Marto, Ana M. Lourenço

**Affiliations:** 1 CIISA—Centre for Interdisciplinary Research in Animal Health, Faculty of Veterinary Medicine, Universidade de Lisboa, 1300-477 Lisbon, Portugal; 2 Associate Laboratory for Animal and Veterinary Sciences (AL4AnimalS), 1300-477 Lisbon, Portugal; 3 Research Institute for Medicine (iMed.ULisboa), Faculty of Pharmacy, Universidade de Lisboa, 1600-277 Lisbon, Portugal; 4 Department of Small Animal Clinical Science, Institute of Infection, Veterinary and Ecological Sciences, University of Liverpool, Leahurst, Neston CH64 7TE, UK

**Keywords:** cannabis, cannabidiol, CBD, canine atopic dermatitis, veterinary medicine

## Abstract

Atopic dermatitis is prevalent in humans (hAD) and dogs (cAD) and profoundly impacts the patients’ quality of life. The increasing number of new drugs in development for atopic dermatitis indicates both the need and potential for precision medicine to generate an optimised benefit–risk therapeutic plan. Cannabidiol (CBD), known for its potential anti-inflammatory and antipruritic properties, shows promise in hAD and cAD management, prompting the exploration of cannabinoids (CBs) and CBD as therapeutic tools. In fact, encouraging results on the benefits of using CBD in cAD have been published, along with safety evaluations that reveal that CBD is generally well tolerated in dogs. However, limited placebo-controlled trials and dosage variations in dogs pose barriers that hinder definitive conclusions. Challenges in product stability, inconsistent formulations, and legal ambiguities highlight the need for standardised CBD-based products for both research and commercial uses. The complex legal landscape further complicates accessibility and regulation. Despite these challenges, CBD is emerging as a potential avenue for cAD management, urging further high-quality research, standardised formulations, and legal clarity. This brief review provides valuable insights into the therapeutic potential of CBs and CBD in cAD, compared to hAD, emphasising the importance of rigorous research and unambiguous regulation for successful integration into veterinary dermatology.

## Simple Summary

This review focuses on studies using cannabinoids (CBs) and cannabidiol (CBD) to treat pruritus and atopic dermatitis in dogs and humans. It provides brief history of the medicinal use of *Cannabis sativa*, its mechanism of action and the effects of CBs on the skin, the pharmacokinetics and safety of CBD in dogs, and the complex legal landscape of cannabis and CBD. It discusses the challenges in maintaining the stability of CBD-based products, including their low solubility in water, oxidative sensitivity, and the impact of storage conditions on their quality and reliability. Most importantly, it highlights the need for properly characterised and controlled products for medical use to ensure their safety. There is limited information on the use of such drugs and their safety in dogs. This review emphasises the need for further research, standardised formulations, and unambiguous regulation to integrate CBD effectively into veterinary dermatology.

## 1. Introduction

Atopic dermatitis is prevalent in humans (hAD) and dogs (cAD). Although it is not life-threatening, atopic dermatitis (AD) profoundly impacts the patients, and in the case of cAD, their owners’, quality of life (QoL), and represents a significant economic burden. Several different treatment options are needed for AD, due to financial constraints, and unpredictable individual responses [1,2]. The steadily increasing number of new drugs in development for hAD and cAD is a good indicator of both the need and potential for precision medicine to generate an optimised benefit–risk therapeutic plan for patients [2]. Cannabidiol (CBD) may represent a new therapeutic tool for cAD, given its potential anti-inflammatory and antipruritic properties, lack of psychoactive effects, and reported minimal side effects [3]. With the growing interest of owners and veterinarians in cannabinoids (CBs), these compounds have been increasingly explored and used in veterinary medicine, especially concerning chronic diseases, such as cAD, where several medications are usually required throughout the pet’s life. CB products are often seen as more natural options and may be preferred over more conventional medications [4,5].

Although CBD seems to be promising as an adjunct treatment for AD and pruritus, there is limited evidence of its efficacy and safety, especially in dogs [4]. And while owners and veterinarians generally hold positive views on CBD usage, many veterinarians express a lack of confidence in their understanding of the benefits and potential risks associated with CB products [6].

In the last ten years, several review articles have been published covering topics such as the use of CBD and other CBs in cutaneous conditions in human [3,7,8,9,10,11,12,13,14,15] and canine medicine [15,16]. To our knowledge, there are still no review papers specifically focused on the use of CBD and other CBs in pruritus and cAD in dogs.

This review will focus on studies with CBs and CBD in pruritus and atopic dermatitis, both in dogs and humans. A brief history, its mechanism of action, CBD pharmacokinetics, the challenges in producing and storing these products, and the “grey” legal area in which CBD falls will also be covered. Articles for this review were selected from ScienceDirect, Pubmed, and Google Scholar databases, using one or a combination of keywords to ensure comprehensive coverage of the topic: cannabis, cannabinoids, cannabidiol, CBD, dog, atopic dermatitis, canine atopic dermatitis, pruritus, eczema, pharmacokinetics, safety, receptors, and stability. Regarding studies on the efficacy of CBs in pruritus and AD, 10 articles were found on hAD, while 6 articles were found on cAD.

## 2. *Cannabis sativa*: A Journey Through Time in Medicine

The first recorded medicinal use of *Cannabis sativa* can be traced back to China around 5000 years ago by Emperor Shén Nóng, who reported the use of *C. sativa* for fatigue, rheumatism, and malaria [17,18,19]. Later, around 1500 BCE, a topical application of *C. sativa* for inflammation was mentioned in the Ebers papyrus in Egypt [18,20]. The medical use of cannabis reached all of Europe and North America, and in the late 19th and early 20th centuries, several scientific articles were published concerning its therapeutic use [21], but the skin was not the usual target.

During the 20th century, the use of *C. sativa* underwent a significant decline. One of the main reasons was the difficulty in standardising cannabis formulations, as the active components were not at that stage identified, and its efficacy was consequently variable [18,21]. Moreover, new medicines and vaccines were developed for many of the therapeutic indications of cannabis. Furthermore, legal constraints limited the medical use and investigation of cannabis [21], and in 1961, the United Nations Single Convention on Narcotic Drugs ultimately led to a worldwide ban on cannabis as a therapeutic tool [18,22]. Despite these obstacles, scientific research continued [23]. The isolation and identification of CBD in 1940 [18,24] and ∆9-tetrahydrocannabinol (THC) in 1964 [18,24,25] opened the door for further research in this field, ultimately leading to the discovery of endocannabinoids and their receptors, confirming the existence of an endocannabinoid system (ECS) in the 1980s [18,26]. Afterwards, there has been a steady increase in the investigation of CBs as therapeutic tools, with several studies being published in the last few years [6,21].

## 3. Understanding the Effects of CBs on Skin: A Focus on CBD

**Figure 1:**
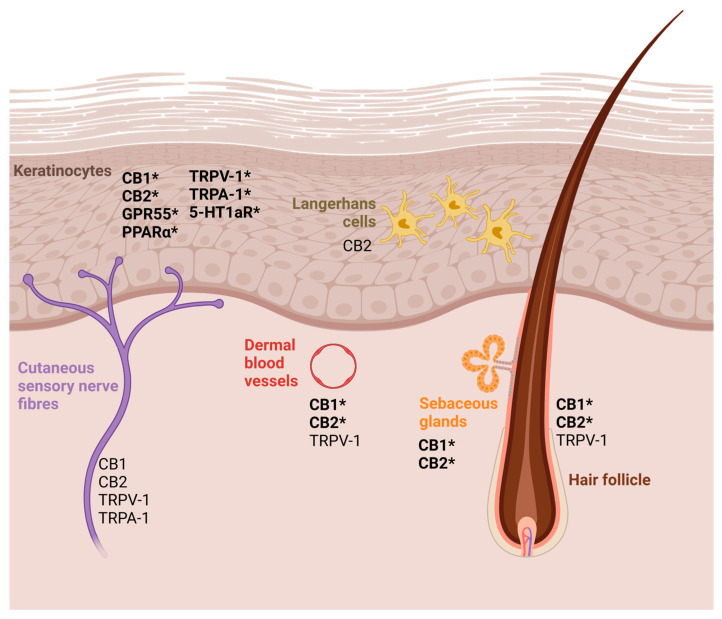
A schematic representation of the CB receptors in the skin, inspired by the diagram by Baswan et al., 2020 [9]. The receptors marked with * have been found in dog skin [31,32]. Although CBD does not appear to have a strong affinity for CB1 and CB2 receptors, it may play a role in other cutaneous CB receptors. CBD may also have an effect by increasing the binding of endocannabinoids (AEA and 2-AG) to CB receptors, including CB1 and CB2, an effect known as the “entourage” effect. CB1 and CB2: cannabinoid receptor 1 and cannabinoid receptor 2; GPR55: G protein-coupled receptor 55; PPARα: peroxisome proliferator-activated receptor α; TRPV-1: vanilloid receptor 1; TRPA-1: transient receptor potential ankyrin 1; 5-HT1aR: serotonin 1A receptor. Created in BioRender. Fernandes, B. (2025). Available online: https://BioRender.com/o61s214 (accessed on 31 January 2025).

CBs comprise a diverse class of compounds that act on cannabinoid receptors (CBRs). There are three main categories of CBs: endocannabinoids (endogenous CBs), synthetic CBs, and phytocannabinoids (those produced by plants). Endocannabinoids are produced in response to stress and tissue damage and play a critical role in regulating cutaneous inflammation and immunity, with the most relevant endocannabinoids being anandamide (AEA) and 2-arachidonoylglycerol (2-AG) [27]. The ECS comprises endogenous endocannabinoids, their receptors (CB1 and CB2), and a complex enzyme and transporter apparatus involved in the synthesis, cellular uptake and release, inter- and intracellular transport, and degradation of endocannabinoids [11]. CB1s are the prevailing CB receptor type in the central nervous system and appear at lower concentrations in the peripheral nervous system [28] and CB2 is the prominent type in the immune system [29]. Both CB1 and CB2 receptors have been found on cutaneous nerve fibres, mast cells, and epidermal keratinocytes of dogs and humans [30,31,32] (Figure 1). More recently, Chiocchetti et al. detected an overexpression of CB2 in keratinocytes from atopic dogs (n = 8), compared to healthy dogs (n = 7); CB1 expression was slightly increased. However, this difference was not considered statistically significant (*p* = 0.24) [32].

Beyond the classical CB1 and CB2 receptor-dependent actions, CBs can also interact with other receptors, including G protein-coupled receptor 55, peroxisome proliferator-activated receptors (PPARs), and transient potential vanilloid receptor (TPVR)-1 [30,33,34]. The latter is expressed on cutaneous sensory nerve fibres, epidermal keratinocytes, dermal blood vessels, and hair follicles [35] (Figure 1). Additionally, CBs can enhance adenosine A2A receptor activity, downregulating over-reactive immune cells and decreasing inflammation in surrounding tissues [36]. For example, palmitoylethanolamide (PEA), an endogenous fatty acid that is not technically a CB as it does not bind to CBRs, enhances binding of endogenous CBs, such as AEA, to CB receptors (CB1 and CB2), the “entourage effect” [37]. AEA downstream signalling conducts the activation of PPAR-α, leading to the inhibition of proinflammatory cytokines (IL-4, IL-5, IFN-γ); the induction, proliferation, and differentiation of keratinocytes; and increased synthesis of lipids, i.e., fatty acids and ceramides, that play an essential role in skin barrier function and integrity [8,11,38].

The most prominent phytocannabinoids within the *C. sativa* plant are THC and CBD [27]. CBD might be of particular interest to the field of dermatology due to its potential antipruritic, anti-inflammatory, and antinociceptive properties, without the psychoactive effects of its counterpart THC [3]. The effects of CBs on the skin are related to their interaction with the skin’s ECS, which plays an essential role in skin homeostasis. Changes in the homeostatic ECS tone are linked to inflammatory diseases, such as AD [39].

Of note, CBD has a low affinity for the endocannabinoid receptors CB1 and CB2, suggesting that its effects are independent of these receptors [40]. CBD can also potentially inactivate the inflammatory cascade by inhibiting nuclear factor (NF)-κB, suppressing IL-6 and IL-17, and upregulating IL-10 via CBR-independent signalling pathways, and modulate pain and itch perception through TRP channels [8]. In 2024, He et al. reversed elevated levels of oxidised lipids resulting from a high-fat, high-cholesterol diet-induced inflammation in mice. Furthermore, they concluded that arachidonic acid metabolism is a central pathway through which CBD exerts its anti-inflammatory effects [41]. Chaoul et al. demonstrated that CBD inhibited mitogen-induced lymphocyte proliferation, reducing the proliferation of T and B cells, without showing cytotoxicity on lymphocytes [42]. Moreover, besides its potential anti-inflammatory and antipruritic effects, CBD has also been proven to have antimicrobial activity against Gram-positive bacteria in vitro, including *Staphylococcus aureus*, *Streptococcus pneumoniae*, and *Clostridioides difficile*, showing excellent activity against biofilms and little propensity to induce resistance, according to Blaskovich et al. [43]. In 2023, Luz-Veiga et al. also reported CBD and cannabigerol activity against biofilm. These CBs inhibited *Staphylococci* adhesion to keratinocytes, and for the first time, minimum inhibitory and lethal concentrations were reported for *Pseudomonas aeruginosa* and *Escherichia coli* [44].

## 4. CBs and CBD: Promising Therapeutic Options for Pruritus and AD in Humans?

Regarding human medicine, few studies have provided evidence of the beneficial effects of CBs on skin diseases.

To our knowledge, no studies are yet available on the effect of orally administered CBs on hAD. Although one study looked at the impact of oral hempseed oil in patients with hAD, hempseed oil only contains residual amounts of CBs, if any. Patients who consumed hempseed oil showed a significant decrease in skin dryness, itchiness (*p* = 0.027), and use of topical medications (*p* = 0.024) compared to those who consumed olive oil. The authors attributed this effect to the high polyunsaturated fatty acid content in hempseed oil, namely linoleic acid (omega-6) and alpha-linolenic acid (omega-3) [45].

Regarding the topical use of CBs for pruritus and hAD, specifically PEA, a CB-like fatty acid, most studies are observational, and there is a lack of a placebo group, so conclusions should be taken cautiously. In most studies, a possible beneficial [46,47,48] effect of CBs could be observed, particularly on pruritus and associated lesions. In an investigator-blinded, split-body, randomised trial, using a PEA cream or a moisturiser, both in association with a topical corticosteroid, PEA cream increased the time to the next flare in AD patients [49]. Visse et al., however, did not find PEA-based lotion to be significantly superior to vehicle lotion in 100 patients with hAD, although some minor effects on pruritus (assessed by a visual analogue scale (pVAS) and by a verbal rating scale) were observed [50].

Even fewer studies are available in human medicine regarding the effects of CBD on pruritus and hAD. The potential beneficial effects on pruritus and/or skin lesions of topical CBD in hAD were observed in all four known studies [42,51,52,53]. Two of the studies are observational and, therefore, not controlled with a placebo group [51,52]. On the other hand, the study by Gao et al. was a randomised, double-blinded, placebo-controlled trial in three groups (CBD and aspartame, CBD only, and placebo); however, only the group with CBD and aspartame had significantly improved skin lesions. There were no significant differences between the CBD-only and the placebo group (*p* = 0.699). Analgesic and anti-inflammatory effects have been reported for aspartame and no group in the study was treated with only aspartame, making it unclear whether the effects were due to aspartame alone or to the combination of aspartame and CBD [53]. In 2024, Chaoul et al. reported a steady decline in pruritus starting from the first week of treatment with the use of a CBD-based cleansing cream and 0.05% clobetasol ointment, compared to a group treated with a CBD-free cleansing oil and the same clobetasol ointment [42]. Nevertheless, the two cleansing formulations differ, one being a cleansing oil and the other a cleansing cream; therefore, the observed results could be either due to CBD or other ingredients in the formulation.

Summarising the results obtained in the studies conducted so far, CBs and CBD may have a potential benefit in the treatment of pruritus and hAD. However, further evidence is needed as studies are mostly pilots, often without a placebo group, and therefore, the outcomes should be interpreted with caution. A summary of the aforementioned studies can be found in Table 1.

## 5. CBs and CBD: Promising Therapeutic Options for Pruritus and AD in Dogs?

In dogs, there are few published studies on the potential efficacy of CBs on skin-related disorders, with only one using a topical approach. In 2019, in a double-blinded, vehicle-controlled, crossover study, Marsella et al. found that a twice-daily inguinal application of a topical compound (WOL067-531, 1% gel) that inhibits endocannabinoid reuptake in dogs, epicutaneously challenged with dust mites (n = 19), significantly decreased pruritus (pVAS) (*p* = 0.032) and dermatitis scores (*p* = 0.029) after 21 days [54]. Furthermore, in an open-label, uncontrolled trial, oral PEA (10 mg/kg/day) administration for 8 weeks appeared to improve pruritus (pVAS) (*p* < 0.0001) and skin lesions (*p* < 0.0001) in dogs with mild cAD (n = 160) [55].

Concerning CBD, in a 2021 randomised, placebo-controlled study assessing daily canine activity of healthy dogs, measured with activity sensors fitted to dogs’ collars, it was reported that despite an oral 4.5 mg/kg/day CBD dose, having no impact on overall daily activity, it significantly reduced scratching behaviour (*p* = 0.030), demonstrating a potential antipruritic effect of this substance [56].

More recently, in 2022, Loewinger et al. carried out a double-blinded, vehicle-controlled, randomised clinical trial to evaluate the effects of oral CBD and cannabidiolic acid (CBDA) in dogs (n = 32) with mild to moderate cAD, and an initial pruritus score of 5.5 ± 1.2 in the CBD group and 5.6 ± 1.4 in the placebo group were found. An improvement in pruritus was reported from day 14 (*p* = 0.04) in dogs taking an equal mix of CBD/CBDA (30/31 mg/mL) in sesame oil capsules with food, 2 mg/kg, twice daily, for 28 days. A third of the treated dogs (6/17) ended the study with a pVAS score ≤ 1.9 cm (with scores in the range of healthy dogs; <2 cm), while no dog in the placebo group (n = 12) achieved a “healthy” score. Other medications were allowed, with explicit inclusion and exclusion criteria, with no alterations throughout the study. Adverse effects were minimal and included lethargy (n = 2 dogs), somnolence (n = 1), decreased aggression (n = 1), increased calmness (n = 3), increased energy/mobility (n = 1), regurgitation (n = 1), increased flatulence (n = 1), and inconsistent appetite (n = 1) in the treatment group. Complete blood count assessment and serum biochemistry evaluations revealed no significant changes between day 0 and day 28 of treatment. While not statistically significant (*p* = 0.28), ALP was elevated (maximum of 1164 U/L) outside of the reference range (23–212 U/L) in 6/17 dogs in the CBD/CBDA treatment. They found no significant differences in the serum levels of the investigated cytokines (MCP-1, IL-6, IL-8, IL-31, IL-34) and the CADESI-04 scores (*p* = 0.51). The authors also measured CBD and CBDA plasma levels and reported modest correlation (r = 0.53; *p* = 0.03) between the decrease in the pVAS score and the serum concentrations of CBD and CBDA. However, the sesame oil capsules did not strictly contain CBD, but also other CBs such as THC and THCA, albeit in low concentrations; this may have influenced the results [57].

Additionally, in a 2022 retrospective study of an oral 10% CBD-containing broad-spectrum hemp oil for cAD (n = 8), Mogi et al. observed an improvement in skin lesions (CADESI-04) and pruritus (pVAS), using an initial dose of 0.07–0.25 mg/kg twice a day (as per the product’s recommendations), with increasing doses of 0.125 mg/kg according to the observed clinical signs (the dose was increased every 4–7 days, when there was no observable changes with the preceding dose), with a final range of 0.14–1.43 mg/kg/day following a twice-daily regimen for at least eight weeks. No adverse events were reported [58]. Nevertheless, the study’s findings should be treated cautiously, given the absence of a control group and the concomitant use of other drugs, without explicit inclusion and exclusion criteria. Furthermore, as a CBD-containing broad-spectrum oil, other CBs could also be present, and in unknown amounts, which might have influenced the reported results.

In 2023, in a prospective randomised study in dogs with cAD (n = 14) with a broad-spectrum CBD oil delivered at a dose of 2.5 mg/kg twice a day for 60 days, Mariga et al. found no significant differences in pruritus (pVAS) (*p* = 0.396), the CADESI-04 score (*p* = 0.654), or histopathological mast cell count in skin biopsies compared to a placebo. The control group, however, was olive oil; hence, the study was not controlled by a vehicle, and no minimum or maximum values for CADESI-04 or pruritus were defined for the inclusion in the study [59].

So far, there are few studies of non-comparable data available regarding CBD in cAD. The CBD-based formulations had different presentations, containing other CBs in smaller amounts, and using different carrier oils. The study designs were also distinct from one another, two being randomised controlled trials (RCTs) and the other a small-scale retrospective study. The doses and time intervals used differed, and both had small sample sizes. On a positive note, in all studies, no severe adverse events were observed; however, only one study investigated haematology and biochemistry blood analyses. Due to the lack of published studies, CBD’s potential therapeutic benefit and apparent tolerability in cAD are currently unknown and further research is warranted. An overview of the referred studies in dogs can be found in Table 2.

## 6. CBD Pharmacokinetics in Dogs

In 2018, Bartner et al. investigated CBD pharmacokinetics in dogs with three different formulations (oral microencapsulated oil beads, CBD-infused oral oil, and transdermal cream) at 10 and 20 mg/kg. The CBD-infused oil formulation provided higher systemic concentrations of CBD), and the most favourable pharmacokinetic profile [60]. That same year, Gamble et al. studied the pharmacokinetics of an oral CBD olive-base oil at 2 mg/kg and 8 mg/kg, with both doses reaching equivalent t_1/2λ_ and similar T_max_ [61]. A subsequent 2020 study by Wakshlag et al. assessed two different oils and one CBD-chew with CBD and CBDA in equal proportions (1 mg/kg each) in healthy dogs. Pharmacokinetic parameters were similar across all formulations at 24 h and 1- and 2-week treatment durations and across the different timepoints [62].

In 2022, Tittle et al. also tested oral CBD in sesame oil or soft gels in dogs (2 mg/kg) with soft gels achieving a higher peak plasma concentration and better acceptability [63]. That same year, Polidoro et al. compared CBD’s intranasal (20 mg), oral (100 mg), and intrarectal (100 mg) administration and observed higher C_max_ in oral administration when compared to the intranasal route. However, no significant differences were found when these values were normalised to dosages of 1 mg/kg due to the dose discrepancy between the two routes of administration and to facilitate the comparison of the systemic exposure, where C_max_ values were divided by 20 and 100 in the intranasal and oral administrations, respectively. While the intranasal delivery enabled quicker absorption, the oral route was preferred for its ease of administration. In contrast, CBD plasma concentrations were undetectable via the intrarectal route [64].

In 2023, Rocca et al. studied CBD’s oral and transmucosal pharmacokinetics (1 mg/kg) in dogs with pain, noting no significant differences in pharmacokinetics between the two administration routes. The strikingly similar plasma concentrations observed in both treatments suggested that CBD absorption through the transmucosal route might be minimal or non-existent, leading to the likelihood that CBD is ingested and then absorbed in the gastrointestinal tract [65].

Limsuwan et al. assessed the pharmacokinetics of four different formulations, including three liquid formulations (5 mg/kg): an oil base (coconut oil), a nanoemulsion base, and a water-soluble base, and a semi-solid form (50 mg per dog). The CBD plasma profile from the water-soluble formulation was comparable to the oil-based group. The nanoemulsion formulation tended to be rapidly absorbed, reaching its peak concentration sooner (T_max_ = 2.00 h) than other formulations. In all four formulations, CBD reached maximum plasma concentrations within 3 h post-administration [66].

CBD also seems to accumulate in the body when administered over prolonged periods. This tendency was evident in studies by Vaughn et al. (2020) and Alvarenga et al. (2023) [67,68]. Vaughn et al., 2020, evaluated the pharmacokinetics of a CBD oil in dogs over 28 days at four different doses and found that plasma concentrations of CBD increased over time. Of the four doses tested (1, 2, 4, 12 mg/kg), significantly higher plasma concentrations were recorded for doses of 4 and 12 mg/kg (*p* < 0.01), with the 4 mg/kg dose being associated with fewer side effects compared to the highest dose [67]. Alvarenga et al. studied the pharmacokinetic parameters of CBD in dogs at doses of 5 and 10 mg/kg over 36 weeks and concluded that the chronic administration of CBD led to dose-proportional accumulations in the body, with higher t_1/2λ_ concentrations [68].

In conclusion, oral administration was commonly chosen in various studies [60,61,62,63,64,65,66,68,69,70], likely due to its ease of administration and widespread use. Oral administration resulted in the highest plasma concentration compared to the alternative tested routes (transdermal, intranasal, intrarectal, and transmucosal). Nevertheless, direct comparisons between these studies are challenging due to the diverse dosages used (1–20 mg/kg), resulting in a wide range of C_max_, T_max_, and t_1/2λ_ values. The number of dogs in the different studies varied between 4 and 12 animals per group, probably dictated by the demanding nature of pharmacokinetic studies, as several blood samples are required. The limited number of animals per study plays a role in the observed outcome variability. The results of the CBD pharmacokinetic studies can be consulted in Table 3.

In general, the higher the doses administered, the higher the plasma concentrations of CBD achieved, at least when comparing identical formulations [60,61,68,69]. However, high doses are also associated with a greater number of side effects, especially when administering CBD chronically; moreover, high doses also entail much higher costs [61], which can be cost-prohibitive both in the academic context and in the day-to-day lives of owners and their pets.

## 7. Can CBs and CBD Be Used Safely in Dogs?

Most information on the safety of CBs in veterinary species is derived from intoxication cases due to accidental exposure to recreational marijuana [71]. Adverse events reported in these cases are associated with all CBs in recreational products, including THC, CBD, and other compounds.

Only a few studies have primarily assessed the safety of CBD alone, in healthy dogs. Most safety data are provided from clinical trials that differ in design, assessed outcomes, formulations, and dosages. Nevertheless, considering most safety studies and clinical trials, CBD appears to be a safe and well-tolerated drug, with only mild adverse events associated with it, most of which are gastrointestinal (nausea, emesis, or diarrhoea) and usually do not require any intervention or interruption of CBD administration [63,67,69,70,72,73,74,75,76].

Another important finding was increased alkaline phosphatase (ALP) (from slight increases to values up to 1511 U/L activity in dogs), highlighting the importance of monitoring liver enzymes, bile acids, and potential drug interactions in dogs taking CBD [61,67,69,70,72,75,76,77]. These increased levels of ALP are not associated with alterations in other liver parameters (ALT, bile acids, GGT, bilirubin). The increase in serum ALP appears to be dose-related and can be observed as early as one week (for higher doses) or two weeks (for lower doses) [67]. Vaughn et al. reported that after two weeks of administration, ALP levels begin to decrease, suggesting an early adaptive response to CBD metabolism [67]. Moreover, according to a 2021 Expert Workshop prepared by the European Society of Toxicologic Pathology, increases in serum ALP activity in the absence of hepatocellular degeneration in dogs could be interpreted as an adaptive response rather than an adverse response to drug exposure [67,78]. The induction of hepatic drug-metabolising enzymes is a plausible explanation for the increased serum ALP observed in most studies. In dogs, serum ALP activity can originate from the liver or bone, or because of endogenous or exogenous corticosteroids [79,80]. Furthermore, in 2022, Bradley et al., in a 6-month, blinded, randomised, placebo-controlled safety trial in 40 healthy dogs with 4 mg/kg broad-spectrum CBD (THC-free) diluted in sunflower oil and manufactured in soft gel capsules, also measured bone-specific alkaline phosphatase (BALP), beyond the measurement of ALP, and reported that BALP was simultaneously elevated, with a significant strong positive correlation between BALP and ALP levels (r > 0.9; *p* < 0.001), suggesting that the observed increase in total ALP could be, at least, partly a consequence of increased osteoblastic activity [75]. In rats, CBD has been shown to improve fracture healing [81] and bone mineral density [82]. Despite these results, some studies have reported non-specific cross-reactivity with other ALP isoenzymes when using immunoassays to evaluate BALP [75,83,84].

The liver plays a central role in drug metabolism, and hepatic cytochrome P450 (CYP) (CYP1, CYP2, and CYP3) enzymes are considered the most critical drug-metabolising enzymes [85,86]. In dogs, some drugs, for example, phenobarbital, have been shown to increase CYP and serum ALP activities with no detectable hepatobiliary obstruction, bone damage, or clinical signs of liver disease [67,87,88]. CBD is metabolised by CYP and functions as an inhibitor of CYP (CYP1A, CYP2C, and CYP3A), which can affect the metabolism and, consequently, increase serum levels of other drugs metabolised by the same CYP pathways, such as antiepileptic drugs, for example [67,89]. Despite the CYP-dependent CBD metabolisation, Doran et al. found no significant pharmacokinetic interactions between CBD and phenobarbital, also metabolised by CYP (CYP2B, CYP2C, and CYP3A), and did not recommend the dose escalation of CBD or adjustment of phenobarbital [90]. The effect of the chronic coadministration of CBD and phenobarbital, however, was not evaluated. Nevertheless, in a pilot study involving client-owned idiopathic epileptic dogs, no significant changes in serum phenobarbital concentrations were found in the seven dogs receiving phenobarbital after 12 weeks of CBD administration [91]. Garcia et al. assessed the safety of a CBD/CBDA-rich hemp extract in dogs with refractory epileptic seizures, 2 mg/kg, every 12 h for 12 weeks, and no differences were observed in serum zonisamide, phenobarbital, and bromide concentrations [77]. In human studies, CBD administration significantly affected serum concentrations of several antiepileptic drugs—clobazam, clonazepam, rufinamide, topiramate, zonisamide, and eslicarbazepine—though no differences were observed in phenobarbital and levetiracetam serum concentrations [92,93].

Regarding the immune response, in 2022, Morris et al. conducted a randomised, placebo-controlled study in healthy dogs (n = 32) immunised with a novel antigen, keyhole limpet hemocyanin (KLH), and taking treats containing CBD in a 5 mg/kg/day dose for 28 days and observed that specific IgG and IgM were similar between treatment groups, suggesting that CBD did not exhibit a humoral immunosuppressive effect when given at that dose and frequency [94].

In addition to the safety data from clinical trials and safety studies, one case report describes a cutaneous drug reaction in a dog, probably caused by a CBD-containing hemp oil for oral administration. The dog presented with pad sloughing and rapidly progressive cutaneous and mucosal ulceration within five days of CBD oil administration. The combination of clinical signs and histopathological findings was consistent with Stevens–Johnson syndrome. All lesions completely resolved after the CBD-containing oil discontinuation. Although CBD was the main active ingredient in the product, other CBs or terpenoids cannot be excluded as possible contributors [95].

The oral doses tested in safety trials varied between 1 and 62 mg/kg [56,57,58,61,63,67,69,70,72,74,76,94]. Considering that most studies opt for approximately 2 mg/kg [56,57,59], the highest dose tested is around 30 times the dose commonly used. If the 2 mg/kg dose is administered twice a day, thus reaching 4 mg/kg a day, the highest dose tested is around 15 times higher than the daily dose usually used. In dogs, available information concerning the safety of CBD-based products for topical application is even scarcer. In the safety study carried out by McGrath, besides oral formulations, a transdermal patch was also tested, with the major adverse effect associated with this formulation being skin erythema at the application site [72]. However, this adverse reaction could be associated with the carrier, as a placebo patch with no CBD was not assessed.

Compared to CBD, THC adverse effects can be more worrying, with the most common ones being lethargy, hypothermia, ataxia, hyperesthesia, muscle tremor, and proprioceptive deficits [69,71]. Therefore, it is of utmost importance that CBD-based products are correctly labelled and characterised to ensure that they have no THC, which can be responsible for more severe and undesired effects, or other CBs or impurities, particularly, as CBs can act synergistically, an effect known as the “entourage effect” [69,96].

## 8. Challenges in Maintaining the Stability of CBD-Based Products

CBD is a highly lipophilic substance with very low solubility in water (0.7 μg/mL) [97]. CBD is oxidatively sensitive and can be affected by the formulation and manufacturing process [98]. Moreover, the conditions under which CBD products are stored can also affect their quality and reliability, with light, temperature, and contact with air all being factors that influence the degradation of CBD [99,100,101].

Even the solvent impacts this compound’s stability [100]. Medium-chain triglyceride (MCT) oil is one of the oils in which CBD is more soluble and is more likely to maintain consistent flavour and visual characteristics over time, with improved bioavailability [102]. According to Pacifici et al., a loss of about 20% of the initial concentration may occur in the first 14 days for CBs when prescribed for medical purposes. Then, for up to one year, CB compounds remain relatively stable [98]. Nevertheless, it is crucial to develop CB-based products with known concentrations, composition, and stability and optimum storage recommendations, for medical use.

## 9. Navigating the Complex Legal Landscape of Cannabis and CBD

The legal status of CBs varies from country to country, including some countries where CBD and THC belong to the same list of prohibited substances, while CBD-based products are legalised in others. Most European countries have legalised the use of cannabis for medical purposes, including Belgium, Croatia, Cyprus, Czech Republic, Denmark, Estonia, Finland, Germany, Greece, Ireland, Italy, Lithuania, Luxembourg, Malta, Moldova, the Netherlands, North Macedonia, Norway, Poland, Portugal, Romania, Switzerland, and the United Kingdom [103]. In Spain, the medical use of cannabis is illegal but decriminalised, and in France, only a few specific preparations are authorised [104,105].

Moreover, although recreational cannabis use is not permitted in Europe, many countries have adopted decriminalisation and limited enforcement policies, where despite the possession of small amounts of cannabis for personal use not being legal, it is not considered a crime [6,103,106].

Regarding CBD, Europe is in a legal grey area. CBD is legal in Austria (but banned from cosmetics and food), Belgium, Bulgaria, Cyprus, Estonia, Finland (by prescription), France, Ireland, Latvia, Lithuania, Luxembourg, Malta (by prescription), Portugal, and Slovenia, provided that the THC content is below 0.2% [103,107]. CBD is illegal in Slovakia, but soon updates in its legal framework are expected [108,109]. In Denmark, CBD-based topical products are legal, if they have THC concentrations below 0.2% and are under medical prescription [103]. In Sweden, CBD-based products are not legalised so far, and cannabis medical use, although legal, is constrained [110].

In the USA, federal and state laws are in conflict, with each state being guided by its own regulations. At the federal level, cannabis is considered a Schedule I substance, thereby making cannabis use a federal offence [111]. However, medical use of cannabis is a reality in some states, even including recreational use in some of them. California was the first state to legalise medical cannabis use in 1996. CBD in the US is listed as a controlled substance in Annex 1 of the Code of Federal Regulations, described as a derivative or component of marijuana. For a product to be FDA-approved, the CBD-based product must contain less than 0.1% THC [103,112].

In Canada, cannabis is legal for medical and recreational use. The Cannabis Act creates a legal framework for controlling the production, distribution, sale, and possession of cannabis across Canada [113].

The heterogeneous legal framework of cannabis and its derivates highlights the need for a speedy solution to the status of CBs.

Since the legislation regarding CBD products is ambiguous [114], companies can produce and distribute CBD products, providing easy access to this compound, leaving consumers with no legal guarantees. Products from uncontrolled sources can be contaminated with harmful substances, namely pesticides, heavy metals, moulds, bacteria, and aflatoxins. CB content of these products may also be unknown, or with concentrations below or above the claimed ones. Several studies have shown that a high number of products containing CBD and other CBs are mislabelled and contain different concentrations than they claim. Bonn-Miller et al. tested the concentration of CBs in 84 products, and concerning CBD, only 30.95% were correctly labelled [115]. In 2018, Pavlovic et al. analysed 14 commercially available CBD preparations, and 9 of them had concentrations that differed significantly from the declared amount [102]. Fernández et al. quantified CBs in samples of 10 oils, and regarding CBD, only 2 were accurately labelled [116]. In 2024, Mouton et al. measured the concentration of CBD in 40 samples of commercially available products, in which only three products (7.5%) were in accordance with the labelling [117]. In 2024, in a study carried out in Portugal by Pires et al., the concentration of CBs in 31 samples was analysed, and concerning CBD, none were correctly labelled [118].

## 10. Conclusions, Challenges, and Future Perspectives

Most studies on the effect of CBs and CBD on pruritus and AD are observational and not placebo-controlled, often with small sample sizes. Concerning CBD, known studies generally use variable/inconsistent doses, and the products used in the available trials often do not exclusively contain CBD but contain other CBs, making it difficult to draw conclusions about the role of CBD alone in conditions such as AD. In dogs, the available studies mainly focus on products for oral administration, and to the authors’ knowledge, to date, there are no published studies in dogs with AD using CBD for topical application for the treatment of this condition. Therefore, further high-quality, randomised, double-blinded, placebo-controlled studies with more robust designs are needed to clarify whether CBD is an advantage as a new therapeutic tool for cAD [10]. Moreover, if CBD indeed has beneficial effects on pruritus and cAD, the most appropriate doses, concentrations, frequency, and route of administration and potential for adverse effects still need to be determined.

The inconsistencies found across studies may also be due to differences between the CB-based products used. Not only because they often contain other CBs that may influence the observed effects but also because the declared number of CBs in various products often differs from the actual amount [102]. Furthermore, since CBD and CBs are unstable compounds, products based on these ingredients need to be stored under specific light and temperature conditions, and particular precautions must be taken when manufacturing these formulations. It is, therefore, essential to develop properly characterised and controlled products for their medical use and to better assess potential efficacy and ensure their safety.

## Figures and Tables

**Table 1:** Summary table of efficacy studies of CBs and CBD in pruritus and AD in humans.

Author(s)	Year	n	Tested Product	Disease	Treatment Regimen	Results	Observations
Ständer et al. [46]	2006	22	PEA cream	Chronic pruritus	Once a day, from 2 weeks to 6 months.	Improved pruritus.	Open observational study. Cream used as antipruritic monotherapy. Quick onset of effect. Pruritus recurrence after discontinuation.
Rosso [49]	2007	43	PEA-containing cream	AD	Twice daily for up to 6 weeks.	PEA-containing cream lengthened the mean time to the next flare.	Pilot study. Investigator-blinded, split-body, randomised trial. PEA cream and placebo used with midpotency corticosteroid. Time-to-flare results from a 12-week follow-up.
Eberlein et al. [48]	2008	2456	0.3% PEA-containing cream	AD	At least twice daily onto the affected skin for 4–6 weeks.	Reduced dryness, excoriation, pruritus, and erythema. Improved sleep quality. In total, 56% of patients stopped topical corticosteroids.	Multinational, multicentre, observational, non-controlled, prospective cohort study.
Callaway et al. [45]	2009	20	Oral hempseed oil	AD	A total of 30 mL orally each day. Two intervention periods of 8 weeks, with a 4-week washout period.	AD patients used hemp seed oil or olive oil. The hemp seed oil group showed a significant decrease in skin dryness, itchiness, and topical medication use compared to the olive oil group.	Twenty-week randomised, single-blind crossover study.
Yuan et al. [47]	2014	60	PEA/AEA emollient	Asteatotic eczema	Twice a day for 28 days.	Improved scaling, dryness, and itching. No difference was found in TEWL.	Randomised, double-blind comparative trial. Emollient with PEA/AEA differs in excipients from product without PEA/AEA.
Visse et al. [50]	2017	100	PEA-enriched lotion	Patients with chronic pruritus due to dry skin	Twice daily for 2 weeks.	Differences in pruritus between groups, not significant. PEA-enriched lotion not superior to base lotion in treating dry, itchy skin.	Randomised, placebo-controlled study.
Palmieri et al. [52]	2019	20	CBD-enriched ointment	Psoriasis and AD and resulting outcome scars	Twice daily for 3 months.	Improved skin parameters and symptoms.	Uncontrolled, retrospective design. Observed results may be influenced by other ointment components.
Gao et al. [53]	2021	57	CBD aspartame combination topical formulation	AD	Twice daily for 14 days.	Topical formulation with CBD and aspartame significantly improved skin lesions. No significant differences between CBD-only and placebo groups.	Double-blinded, placebo-controlled, randomised study. Three groups: CBD and aspartame, only CBD, and placebo. No aspartame-only control complicates attributing effects to aspartame or CBD–aspartame combination.
Maghfour et al. [51]	2021	14	1% CBD-infused gel	AD	Variable frequency of application for 14 days.	Improvement in pruritus and skin lesions.	Small observational study. Clinicians were not blinded. No control groups.
Chaoul et al. [42]	2024	28	CBD-based cleansing cream (0.05 mg/mL)	AD	Cleansing oil or a CBD-based cleansing cream, daily for 5 weeks.	Both treatments induced a reduction in the VAS score after 5 weeks. The CBD group also had a steady decline in the VAS score starting from the first week.	Prospective, controlled study. The two cleansing formulations were different in their excipients; the observed results could be either due to CBD or other ingredients in the formulation itself. All patients applied topical clobetasol and received oral fenofenadine, and therefore a reduction in the VAS score was expected in both groups.

**Table 2:** Summary table of efficacy studies of CBs and CBD in pruritus and cAD in dogs.

Author(s)	Year	n	Tested Product	Disease	Treatment Regimen	Results	Observations
Noli et al. [55]	2015	160	Oral ultra-micronised PEA	cAD	A total of 10 mg/kg once daily for 8 weeks.	Reduced pruritus and skin lesions. Improved quality of life.	Open-label and uncontrolled-designed multicentre study.
Marsella et al. [54]	2019	19	A 1% topical compound that inhibits endocannabinoid reuptake, 2 mg/cm^2^	cAD	Twice daily for 21 days.	Significantly decreased pruritus and dermatitis scores.	Double-blinded, vehicle-controlled, crossover study. Possible underappreciation of tested formulation’s benefit. Pruritus evaluated before, not after, product application.
Morris et al. [56]	2021	24	CBD treats	Healthy dogs	A total of 0 (placebo), 2.5 mg (LOW), and 5 mg (HIGH) CBD/kg/day, split between 2 treatments. After 7 days of treatment acclimation, data were collected for 14 days.	No impact on overall daily activity. Reduced scratching behaviour in healthy dogs.	Randomised complete block design. Trace THC was present in both LOW and HIGH treatments.
Loewinger et al. [57]	2022	32	Oral CBD and CBDA sesame oil	cAD	A total of 2 mg/kg twice daily, with a meal and for 28 days.	Pruritus improvement. No significant differences in investigated cytokine levels and CADESI scores.	Double-blinded, placebo-controlled, randomised trial. Allowed other medications with defined criteria. Modest correlation between decreased pVAS score and CBD/CBDA serum concentrations. Sesame oil capsules contained other CBs (THC, THCA) in low concentrations.
Mogi et al. [58]	2022	8	Oral 10% CBD-containing broad-spectrum hemp oil	cAD	A total of 0.07–0.25 mg/kg twice a day, with increasing doses according to the observed clinical signs, with a final range of 0.14–1.43 mg/kg/day, for at least 8 weeks.	Improvement in skin lesions and pruritus.	Retrospective study. No control groups. Concomitant use of other drugs without explicit criteria. CBD-containing broad-spectrum oil may include other CBs in unknown amounts.
Mariga et al. [59]	2023	14	Full spectrum with a higher concentration of CBD at 1500 mg, in a ratio of 21:1 for CBD: THC	cAD	A total of 2.5 mg/kg twice a day for 60 days.	No significant changes in skin lesions, pruritus, or mast cell count.	Control group with olive oil (different vehicle). No specified maximum or minimum CADESI values for selection or exclusion.

**Table 3:** Summary table of CBD pharmacokinetic parameters in dogs: maximum serum concentration (C_max_), time to reach maximum serum concentration (T_max_), and half-life (t_1/2λ_).

Authors	Year	n	Tested Product(s)	Dose	C_max_	T_max_	t_1/2λ_
Bartner et al. [60]	2018	5/group	Three different formulations (oral microencapsulated oil beads, CBD-infused oral oil, transdermal cream)	10 and 20 mg/kg	10 mg/kg: 625.3 ng/mL 20 mg/kg: 845.5 ng/mL	-	10 mg/kg: 199.7 min 20 mg/kg: 127.5 min
Gamble et al. [61]	2018	4/group	Oral CBD olive-based oil	2 mg/kg and 8 mg/kg	2 mg/kg: 102 ng/mL	2 mg/kg: 1.5 h	2 mg/kg: 4.2 h
					8 mg/kg: 591 ng/mL	8 mg/kg: 2.0 h	8 mg/kg: 4.2 h
Wakshlag et al. [62]	2020	6/group	Two oils (MCT and sesame oil) and one chew	1 mg/kg	MCT oil: 145 ng/mL	MCT oil: 1.5 h	MCT oil: 4.1 h
					Sesame oil: 124 ng/mL	Sesame oil: 2.0 h	Sesame oil: 4.4 h
					Chew: 226 ng/mL	Chew: 2.5 h	Chew: 3.8 h
Vaughn et al. [67]	2020	4/group	CBD MCT oil	1, 2, 4, and 12 mg/kg, single dose and after 28 days	1 mg/kg: 30 ng/mL	1 mg/kg: 4.5 h	1 mg/kg: 5.6 h
					2 mg/kg: 26 ng/mL	2 mg/kg: 3.5 h	2 mg/kg: 9.3 h
					4 mg/kg: 130 ng/mL	4 mg/kg: 3.5 h	4 mg/kg: 5.4 h
					12 mg/kg: 201 ng/mL	12 mg/kg: 4.5 h	12 mg/kg: 7.2 h
					1 mg/kg, 28 d: 53 ng/mL	1 mg/kg, 28 d: 3.0 h	1 mg/kg, 28 d: 24.6 h
					2 mg/kg, 28 d: 115 ng/mL	2 mg/kg, 28 d: 2.3 h	2 mg/kg, 28 d: 19.0 h
					4 mg/kg, 28 d: 194 ng/mL	4 mg/kg, 28 d: 3.3 h	4 mg/kg, 28 d: 22.7 h
					12 mg/kg, 28 d: 285 ng/mL	12 mg/kg, 28 d: 5.8 h	12 mg/kg, 28 d: 13.8 h
Tittle et al. [63]	2022	8/group	Oral CDB in sesame oil (SO) and in soft gels (SG)	2 mg/kg	SO: 184.5 ng/mL	SO: 1.4 h	SO: 3.4 h
					SG: 267.6 ng/mL	SG: 1.1 h	SG: 2.2 h
Polidoro et al. [64]	2022	6/group	Intranasal (IN), oral, and intrarectal (IR) CBD	IN (20 mg)	Oral: 216.76 ng/mL	Oral: 3.50 h	Oral: 15.65 h
				Oral (100 mg)	IN: 27.96 ng/mL	IN: 0.49 h	IN: 7.02 h
				IR (100 mg)	IR: undetected	IR: undetected	IR: undetected
Rocca et al. [65]	2023	12	Oral and transmucosal (TM) CBD	1 mg/kg	Oral: 206.77 ng/mL	Oral: 2.17 h	Oral: 2.67 h
					TM: 200.33 ng/mL	TM: 2.17 h	TM: 2.62 h
Alvarenga et al. [68]	2023	6/group	CBD MCT oil	5 and 10 mg/kg single dose and after 36 weeks	5 mg/kg: 441 ng/mL	4 mg/kg: 3.5 h	5 mg/kg: 8.8 h
					10 mg/kg: 880 ng/mL	12 mg/kg: 4.5 h	10 mg/kg: 12.6 h
					5 mg/kg, 36 w: 616 ng/mL	5 mg/kg, 36 w: 2 h	5 mg/kg, 36 w: 30.6 h
					10 mg/kg, 36 w: 1746 ng/mL	10 mg/kg, 36 w: 3 h	10 mg/kg, 36 w: 26.9 h
Limsuwan et al. [66]	2024	8/group	Three liquid formulations (coconut (CO) oil, nanoemulsion (NE), and water bases (W)) and one semi-solid (SS) form	Liquid forms (5 mg/kg) SS (50 mg/dog)	CO: 270.10 μg/L	CO: 3.21 h	CO: 8.47 h
					NE: 175.35 μg/L	NE: 2.00 h	NE: 10.19 h
					W: 314.30 μg/L	W: 2.58 h	W: 10.23 h
					SS: 92.29 μg/L	SS: 2.83 h	SS: 9.56 h

## Data Availability

No new data were created or analyzed in this study. Data sharing is not applicable to this article.
